# Skin cancers among Albinos at a University teaching hospital in Northwestern Tanzania: a retrospective review of 64 cases

**DOI:** 10.1186/1471-5945-12-5

**Published:** 2012-06-08

**Authors:** Joseph B Mabula, Phillipo L Chalya, Mabula D Mchembe, Hyasinta Jaka, Geofrey Giiti, Peter Rambau, Nestory Masalu, Erasmus Kamugisha, Ssentongo Robert, Japhet M Gilyoma

**Affiliations:** 1 Department of Surgery, Catholic University of Health and Allied Sciences-Bugando, Mwanza, Tanzania; 2 Department of Surgery, Muhimbili University of Health and Allied Sciences, Dar Es Salaam, Tanzania; 3 Department of Internal Medicine, Catholic University of Health and Allied Sciences-Bugando, Mwanza, Tanzania; 4 Department of Pathology, Catholic University of Health and Allied Sciences-Bugando, Mwanza, Tanzania; 5 Department of Oncology, Catholic University of Health and Allied Sciences-Bugando, Mwanza, Tanzania; 6 Department of Molecular biology and Biochemistry, Catholic University of Health and Allied Sciences-Bugando, Mwanza, Tanzania; 7 Department of Plastic and Reconstructive Surgery, Mulago Hospital Complex, Kampala, Uganda

**Keywords:** Skin cancers, Albinism, Pattern, Treatment outcome, Challenges of care, Tanzania

## Abstract

**Background:**

Skin cancers are a major risk associated with albinism and are thought to be a major cause of death in African albinos. The challenges associated with the care of these patients are numerous and need to be addressed. The aim of this study was to outline the pattern and treatment outcome of skin cancers among albinos treated at our centre and to highlight challenges associated with the care of these patients and proffer solutions for improved outcome.

**Methods:**

This was a retrospective study of all albinos with a histopathological diagnosis of skin cancer seen at Bugando Medical Centre from March 2001 to February 2010. Data collected were analyzed using descriptive statistics.

**Results:**

A total of 64 patients were studied. The male to female ratio was 1.5:1. The median age of patients was 30 years. The median duration of illness at presentation was 24 months. The commonest reason for late presentation was financial problem. Head and the neck was the most frequent site afflicted in 46(71.8%) patients. Squamous cell carcinoma was the most common histopathological type in 75% of cases. Surgical operation was the commonest modality of treatment in 60 (93.8%) patients. Radiotherapy was given in 24(37.5%) patients. Twenty-seven (42.2%) of the patients did not complete their treatment due to lack of funds. Local recurrence following surgical treatment was recorded in 6 (30.0%) patients. Only thirty-seven (61.7%) patients were available for follow-up at 6–12 months and the remaining patients were lost to follow-up.

**Conclusions:**

Skin cancers are the most common cancers among albinos in our environment. Albinism and exposure to ultraviolet light appears to be the most important risk factor in the development of these cancers. Late presentation and failure to complete treatment due to financial difficulties and lack of radiotherapy services at our centre are major challenges in the care of these patients. Early institution of preventive measures, early presentation and treatment, and follow-up should be encouraged in this population for better outcome.

## Background

Albinism is a genetically inherited disorder characterized by hypopigmentation of the skin, hair and eyes due to a reduced or lack of cutaneous melanin pigment production [1]. Generally, there are two principal types of albinism, *oculocutaneous*, affecting the eyes, skin and hair, and *ocular* affecting the eyes only [1,2]. The mode of inheritance of albinism is thought to vary, depending on the type. The oculocutaneous type is considered autosomal recessive, and the ocular variant sex-linked [1-4]. Oculocutaneous albinism exists in four forms. One form involves the tyrosinase gene (OCA1), whereas the other form (OCA2) has recently been associated with alterations of the P gene on chromosome 15. The other two forms include OCA3 due to TYRP1 mutations and OCA4 due to SLC45A2/MATP [5]. OCA 2 is about twice as common as OCA1 in African and African-American populations [1,5].

Albinism has a worldwide distribution and tends to affect people of all ethnic backgrounds; its frequency worldwide is estimated to be approximately 1 in 20,000 in most populations [4-6] and in Africa, incidences ranging from 1 in 2,700 to 1 in 10,000 people have been reported in various studies [4,5,7-11] with the highest incidence of 1 in 1,000 people in Zimbabwe [12]. In Tanzania the frequency of albinism has been estimated to be approximately 1 in 2,500 [9-11].

Melanin is a photo protective pigment, protecting the skin from the harmful effects of ultraviolet radiation. Its deficiency in people with albinism predisposes them to the harmful effects of ultraviolet radiation exposure, resulting in issues such as photophobia, decreased visual acuity, extreme sun sensitivity, and skin cancers [11,13]. High levels of exposure to ultraviolet radiation increase the risk of all three major forms of skin cancer and are responsible for the anatomical site distribution [14]. No use of protection for the skin increased the risk of skin cancer in these patients.

The head and the neck is the site most commonly affected and squamous cell carcinoma has been reported to be the commonest skin malignancy seen in albinos [7,8]. In Africa the incidence of squamous cell carcinoma in the general population ranges from 7.8 to 16% of all diagnosed skin malignancies [4,7]. In the African albino, the risk of developing these malignancies in comparison to the general population has been reported to be as high as up to 1000 fold [4].

The management of skin cancers among albinos in resource-limited countries like Tanzania poses major therapeutic challenges which need to be addressed. Late presentation with advanced lesion coupled with lack of therapeutic facilities such as radiotherapy services are among the hallmarks of the disease in developing countries. The outcome of treatment of skin cancers among albinos in most developing countries has been poor because the majority of these patients present late to the hospital with advanced stage. This is partly due to paucity of local data regarding this condition and lack of community awareness on the importance of early reporting to hospital for early diagnosis and treatment. This study was conducted to describe the pattern and treatment outcome of skin cancers among albinos treated at our centre and to highlight challenges associated with the care of these patients and proffer solutions for improved outcome.

## Methods

### Study design and setting

This was a retrospective study of all the albinos with a histopathological diagnosis of skin cancer treated at Bugando Medical Centre (BMC) between March 2001 and February 2010. Bugando Medical Centre is a tertiary care and teaching hospital for the Catholic University of Health and Allied Sciences-Bugando and has a bed capacity of 1000. It is one of the four largest referral hospitals in the country and serves as a referral centre for tertiary specialist care for a catchment population of approximately 13 million people from Mwanza, Mara, Kagera, Shinyanga, Tabora and Kigoma. The hospital has a newly established Oncology department which provides care for all patients with histopathologically proven cancers including skin cancers. However, unfortunately the department does not provide radiotherapy services at the moment due to lack of this facility at our centre. As a result patients requiring this modality of treatment have to travel long distances to receive radiotherapy at the Tanzania Tumor Centre located a considerable distance from the study area.

### Study subjects

The subjects in this study included all the albinos with a histopathological diagnosis of skin cancer treated at our hospital during the period studied. Patients with incomplete data were excluded from the study.

The details of patients were collected from patients’ files kept in the Medical record department, the surgical wards, operating theatre and histopathology laboratory. Information was collected using a preformed questionnaire. Data included in the questionnaire were demographic data (age, sex, and occupation), duration of illness, anatomical site, histopathological types, treatment modalities, outcome (i.e. postoperative complications, length of hospital stay and mortality) and follow-up.

### Statistical analysis

Statistical data analysis was done using SPSS software version 17.0 (SPSS, Inc, Chicago, IL). Data was summarized in form of proportions and frequency tables for categorical variables. Continuous variables were summarized using means, median, mode and standard deviation. P-values were computed for categorical variables using Chi – square (χ^2^) test and Fisher’s exact test depending on the size of the data set. Independent student t-test was used for continuous variables. A p-value of less than 0.05 was considered to constitute a statistically significant difference.

### Ethical consideration

Ethical approval to conduct the study was obtained from the CUHAS/BMC Joint Institutional Ethic Review Committee before the commencement of the study.

## Results

### Socio-demographic characteristics

During the period under review, a total of 486 histopathologically confirmed skin cancers were registered. Of these, sixty-four (13.2%) were albinos which formed the study population, all of them were of African heritage. There were 38 (59.4%) males and 26 (40.6%) females with a male to female ratio of 1.5:1. The ages ranged from 18 to 58 years with a mean and median age of 29.9 ± 8.6 and 30 years respectively. The majority of patients, 58(90.6%) were aged below 40 years. The modal age group was 21–30 years accounting for 45.3% of cases (Table 1). Fifty-four (84.4%) of the patients were outdoor workers involved in semi-skilled and unskilled labour and majority of them, 60 (93.8%) came from the rural areas located a considerable distance from Mwanza City.

**Table 1:** Age group distribution

**Age group (in years)**	**Number of patients**	**Percentage**
11-20	5	7.8
21-30	29	45.3
31-40	24	37.5
41-50	4	6.3
51-60	2	3.1
**Total**	**64**	**100**

### Clinical presentation

The duration of illness ranged from 2 to 56 months (mean 26.16±10.23, median = 24 months) and the majority of patients, 48 (75%) presented between 2 and 24 months of onset of illness. Most of the patients presented late with ulcers/fungating fleshy lesions, with a median time at presentation of 24 months. The reasons for late presentation are shown in Table 2.

**Table 2:** Reasons for late presentation

**Reasons for late presentation**	**Number of patients**	**Percentage**
Financial problem	60	93.8
Treated at peripheral hospitals	46	71.9
Long distance to health facilities	40	62.5
Self medications at home	32	50.0
Treated by traditional healers	27	42.2
No reasons documented	19	29.7

**Figure 1:**
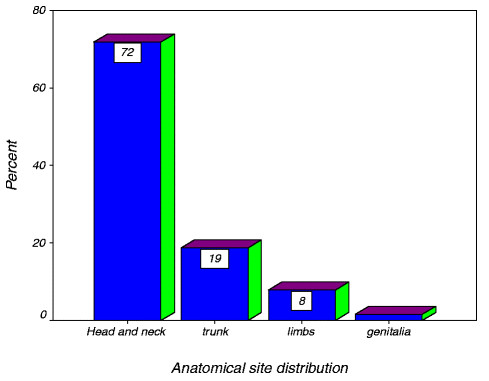
Distribution of study population according to anatomical site affected.

Head and the neck was the most frequent site afflicted in 46(71.8%) patients, followed by the trunk in 12 (18.8%) patients. The limbs and genitalia were least affected in 5(7.8%) and 1(1.6%) patients respectively (Figure 1).

The median tumor size at presentation was 7 cm (range 2 to 18 cm) and the vast majority of patients, 52(81.3%) presented with large tumors of more than 5 cm in diameter. Most patients, 52 (81.3%) had no evidence of metastases. Lymph node metastasis at the time of diagnosis was recorded in 8(12.5%) patients. Distant metastasis was recorded in six (9.4%) patients and occurred mostly in the lungs, liver, bone and brain in 3 (50.0%) and 1(16.7%) patient each respectively.

### Histopathological type

Squamous cell carcinoma (SCC) was the most common histopathological type in 48(75.0%) patients. This was followed by Basal cell carcinoma (BCC) and Malignant melanoma (MM) in 15 (23.4%) and 1(1.6%) patients respectively. Anatomical distribution of lesions according to histopathological types is shown in Table 3. The histopathological grade of these tumors were well, moderately and poorly differentiated in 18(28.1%), 20 (31.3%) and12 (18.8%) patients respectively. The histopathological grade was not documented in 14 (21.9%) of cases. HIV status was known in 42 (65.6%) patients; of these, four (6.2%) were HIV positive. Of the HIV positive patients, three patients had SCC and one patient had BCC. Secondary infection of their lesions was sometimes noted and the common isolates were *Staphylococcus aureus*, *Pseudomonas*, and *Proteus*. We could not have the ability to determine the number of patients that were OCA1 or 2.

**Table 3:** Anatomical distribution of lesions versus histopathological types (N = 64)

**Anatomical site**	**Histopathological type**			**Total (N/%)**
	**SCC (N/%)**	**BCC (N/%)**	**MM (N/%)**	
Head & neck	31 (48.4)	15 (23.4)	-	46 (71.8)
Trunk	12 (18.8)	-	-	12 (18.8)
Limbs	4 (6.2)	-	1 (1.6)	5 (7.8)
Genitalia	1 (1.6)	-	-	1 (1.6)
Total	48 (75.0)	15 (23.4)	1 (1.6)	64 (100)

Keys: SCC = Squamous cell carcinoma, BCC = Basal cell carcinoma, MM = Malignant melanoma.

### Treatment modalities

The majority of patients, 58 (90.6%) required hospitalization for their treatment and the remaining 6(9.4%) were treated as outpatients. Surgical operation was the commonest modality of treatment in 60 (93.8%) patients as shown in Table 4. This was usually combined with adjuvant or palliative radiotherapy for recurrent lesions as well as deep seated inoperable lesions. In this study, radiotherapy was given in 24(37.5%) patients, even though 13 more patients could have benefited from radiotherapy.

**Table 4:** Types of surgical procedures performed (N = 60)

**Surgical procedures performed**	**Frequency**	**Percentage**
*Wide local excision*	52	86.7
Simple	42	80.8
*With skin grafting*	6	11.5
*With flaps*	4	7.7
Limb amputation	2	3.3
Lymph node dissection	1	1.7
Biopsy only	4	6.7

Twenty-seven (42.2%) of the patients did not complete their treatment or were lost to follow up shortly after commencement of treatment. Thirteen (48.1%) of these were patients requiring adjuvant radiotherapy. Most had complained of financial problem at the time of referral for radiotherapy.

### Outcome and follow up of patients

Post-operative complications were recorded in 20 (31.3%) patients. Of these, surgical site infection was the most common post-operative complication accounting for 55.0% of cases (Table 5). The length of hospital stay for in-patients ranged from 8 to 52 days with a median of 12 days. Four patients died in hospital giving a mortality rate of 6.3%. Mortality was significantly associated with delayed presentation (*P* = 0.018), HIV status (*P* = 0.001) and presence of complications (*P* = 0.015). Of the survivors, fifty-one (85.0%) healed with no local recurrence after 12-24 months of follow-up. Only thirty-seven (61.7%) patients were available for follow up at 6–12 months and the remaining patients lost to follow-up.

**Table 5:** Postoperative complications (N = 20)

**Postoperative complications**	**Frequency**	**Percentage**
Surgical site infection	11	55.0
Local recurrence	6	30.0
Loss of skin grafting	2	10
Loss of flaps	1	5.0

## Discussion

In this review, skin cancers in albinos accounted for 13.2% of total skin cancers which is comparable to a Nigerian study by Asuquo and Ebughe [15]. Kromberg *et al*. [8], reported that 23.4 percent of albinos developed skin cancer out of 111 albinos studied in South Africa. These differences reflect geographical variations in risk of environmental exposure to ultraviolet light exposure which is the major etiological factor for skin cancers. The figures in our study may actually be an underestimate and the magnitude of the problem may not be apparent because of retrospective nature of this study.

In albinos there is a defect in the synthesis of tyrosinase, which catalyses hydroxylation of the melanin precursor tyrosine to dioxyphenylalanine [16]. As a consequence these persons lack the protective effect of melanin against ultraviolet radiation damage. The Tanzanian albino is particularly predisposed to skin cancer, because of the proximity of this country to the equator and consequently high intensity of ultraviolet light [10,13].

Skin cancers are generally commoner in the middle aged and elderly. In albinos however these cancers are known to present earlier [4,7,9,17]. Ademiluyi and Ijaduola [18] reported that black patients presented between the 3rd and 4th decades, whereas the albinos presented a decade earlier. Yakubu and Mabogunje [7] in northern Nigeria reported that albinos seldom live more than 30 years. In his review of 1000 Nigerian albinos, Okoro [13] found none above the age of 20 to be free of solar induced pre-malignant or malignant skin lesions. Launde *et al.*[10] in their review of 350 albinos in Dar-es-Salaam reported a similar finding in which the peak age of patients with advanced skin cancers was the 4th decade of life. In the present study, more than ninety percent of patients were aged below 40 years. The reason for this age differences remains unclear. The finding that the majority of patients were aged below 40 years calls for early institution of preventive modalities in albinos.

In this study, male patients were more affected than females. This is in keeping with other studies done elsewhere [7,9,19], but at variance with one Nigerian study which reported no gender difference giving a male to female ratio of 1:1 [4]. The male preponderance in this study could be explained by the fact that men tend to spend more time outdoor in farming activities and other types of outdoor work and hence they are more likely to be exposed to high intensity of ultraviolet light which is the major etiological factor for skin cancers in albinos.

Most of the patients in our study came from the rural areas located a considerable distance from Mwanza City. Similar observation was also reported in other African studies [4,7,9,19]. This observation may explain the reason for late presentation to hospital in the majority of cases. Delayed presentation for treatment is still a common feature in most patients in Africa, as reported by other studies [4,9,10,15,19]. Late presentation was a prominent feature in this study. The average duration of symptoms of 24 months among these patients and the advanced nature of the presenting lesions suggest serious delays in seeking proper medical attention. Financial problem was the main reasons for this. Some however presented early to a healthcare facility, but were offered inadequate or ineffective forms of treatment, only to be referred late.

In this study, the head and neck was the most frequent anatomical site affected whereas the limbs and genitalia were least affected. Similar anatomical site distribution was also reported in other studies [4,7-9,15,19], and is similar to the pattern of non-melanotic skin cancers seen in non albinos of Caucasian descent. As in the Caucasians, sun exposure is thought to be the major aetiological factor for cutaneous cancers in albinos [7,8,20,21]. The occurrence of these tumors in sun-exposed parts of the body suggests the role of solar radiation as a risk factor in skin cancer in albinos and may also be responsible for this pattern of distribution. In this study, we could not establish the possible risk factor(s) in lesions of the trunk, limbs and genitalia.

In our series, distant metastasis was recorded in only 9.4% of patients which is in keeping with other studies done elsewhere [4,7-9]. The low incidence of distant metastasis observed in this study can be explained in part by the fact that squamous cell carcinomas arising in sun damaged areas have a lower incidence of metastasis than do carcinomas arising from chronic ulcerations or de novo [4,6,9].The reason for this observation is not known.

In agreement with other studies [9,10], the most common histopathological pattern in the present study was Squamous cell carcinoma (75.0%), followed by Basal cell carcinoma (23.4%) and Malignant melanoma (1.6%). Unlike in whites where basal cell carcinoma is by far the commonest histological variant, [7,8,20,21] in albinos, as was seen in this study, the squamous cell variety appears to be commoner [4,7-10,20]. The occurrence of malignant melanomas in albinos has been reported in literature to be rare [22]. Datubo-Brown in Nigeria [23], and Kromberg in southern Africa [8], reported the absence of melanoma in their studies and highlighted the rarity of this tumor in albinos. Luande *et al *[10] in Tanzania reported SCC in 29 out of 33 patients with one melanoma and three basal cell carcinoma patients. As in other reports from Africa [4,7-9,21], no cases of Kaposi sarcoma were seen in our albino patients.

Surgery has been reported to be the mainstay of treatment of the majority of skin cancers in albinos [4,24]. Adequate surgical resection is most important to prevent local recurrence. Good results can be obtained with radical surgery and optimal surgical margins along with reconstructive procedure when needed. In the present study, wide local excision was the most common surgical procedure performed in 93.8% of cases. However, with these patients presenting late and majority of the lesions affecting the head and neck, defects following resection were usually complex and affected multiple aesthetic units and or major proportions of single aesthetic units. Reconstruction was therefore often complex and multi-staged. Radiotherapy was given in only 37.5% of all cases requiring this modality of treatment. Radiotherapy for skin cancers in albinos is recommended in the following conditions: (1) inoperable lesions, or those for which an effective operation is unreasonable, (2) multiple lesions, and (3) in patients with medical contraindications to surgery. In our study, radiotherapy was required in patients with advanced disease and those with SCC and BCC located in areas such as the nose, lip, eyelid and canthus, where surgery is either technically difficult or likely to yield poor cosmesis palliation. Since radiotherapy is not available at our centre, patients requiring radiotherapy had to go to Oncological centre (located a considerable distance from Mwanza city) for such treatment and the logistical arrangements for this are difficult, expensive and slow and as a result of this, only 37.5% of cases had radiotherapy.

In the present study, more than forty percent of patients did not complete their treatment or were lost to follow up shortly after commencement of treatment and most of these were patients requiring adjuvant or palliative radiotherapy. This observation is in keeping with other African studies [4,7,8]. Failure to complete treatment in our patients can be explained by the fact that radiotherapy is not available in our centre and therefore patients requiring this form of treatment had to travel long distances to receive radiotherapy at the Oncological centre and because of lack of funds at the time of referral for radiotherapy in the majority of patients, only 37.5% of patients were able to travel and received this form of treatment. However, despite this treatment challenges, more than eighty percent of survivors healed completely with no local recurrences after 12-24 months of follow- up. In the present study, local recurrence following surgical treatment was recorded in 30.0% of cases which is higher rate than that reported by other authors [7,8,19,24]. High recurrence rate in our study is attributed to delayed presentation and failure to complete treatment.

The overall mortality rate in this study was 6.3% and it was significantly associated with delayed presentation, HIV status and presence of complications. Addressing these factors responsible for high mortality in our patients is mandatory to be able to reduce mortality associated with this disease.

From available reports, skin cancers in albinos are preventable [4,6,13,21]. There is therefore a need for early institution of skin protective measures in these patients and these include protective clothing, sun-screening agents, indoor occupations, and early presentation and treatment of skin cancer. Many albinos particularly in the rural regions of Tanzania are unaware that these devastating skin changes are due to exposure to sunlight.

Despite its retrospective nature, the present study described the pattern of skin cancers seen in Albinos, and highlighted and addressed challenges faced in the care of these patients in our environment.

## Conclusions

Squamous cell carcinoma of the head and neck region are the most frequently diagnosed cancers among albinos in our environment. Albinism and exposure to ultraviolet light appears to be the most important risk factor in the development of skin cancers. Late presentation and failure to complete treatment due to financial problem and lack of radiotherapy services at our centre are major challenges in the care of these patients. To address this, we recommend that the public receive education on early institution of preventive measures. Registering all albinos early in life, educating them to prevent the damaging effect of the sun (protective clothing, sun-screening agents and indoor occupations), detecting and treating pre-malignant and malignant lesions are of great importance in this part of the world. Providing free annual skin check up would improve early detection and treatment, hence reducing the morbidity and mortality of skin cancers in these patients. There is a need for the government to provide treatment funds for these poor patients as a significant number of patients were unable to complete treatment due to lack of funds. Establishment of radiotherapy services at our centre is highly recommended.

## Competing interests

The authors declare that they have no competing interests.

## Authors’ contributions

JBM conceived the study, participated in the design and coordination of the study and drafted the manuscript. PLC contributed in study design, literature search, data analysis, manuscript writing, editing and submission of the manuscript. MDM, HJ and GG participated in study design, data analysis, manuscript writing & editing. PR participated in study design, reviewing the histopathological data, manuscript writing & editing. NM, EK and RS participated in data analysis and manuscript writing. JMG supervised the study and contributed in data analysis, manuscript writing & editing. All the authors read and approved the final manuscript.

## Pre-publication history

The pre-publication history for this paper can be accessed here:

http://www.biomedcentral.com/1471-5945/12/5/prepub
